# Obesity is associated with improved postoperative overall survival, independent of skeletal muscle mass in lung adenocarcinoma

**DOI:** 10.1002/jcsm.12956

**Published:** 2022-02-25

**Authors:** Ji Hyun Lee, Young Cheol Yoon, Hyun Su Kim, Min Jae Cha, Jae‐Hun Kim, Kyunga Kim, Hye Seung Kim

**Affiliations:** ^1^ Department of Radiology Samsung Medical Center, Sungkyunkwan University School of Medicine Seoul Republic of Korea; ^2^ Department of Radiology Chung‐Ang University Hospital, Chung‐Ang University College of Medicine Seoul Republic of Korea; ^3^ Biomedical Statistics Center Research Institute for Future Medicine, Samsung Medical Center Seoul Republic of Korea

**Keywords:** Obesity, Body mass index, Skeletal muscle, Sarcopenia, Lung adenocarcinoma

## Abstract

**Background:**

Although the obesity paradox is a topic of immense interest for oncologists and epidemiologists, the mechanism underlying this unexpected benefit of obesity is poorly understood. We explored the prognostic value of obesity and its association with skeletal muscle mass.

**Methods:**

This retrospective study evaluated the data of patients who underwent surgical excision for lung adenocarcinoma between January 2011 and December 2015. Body mass index was categorized according to the criteria of the Asia‐Pacific classification. Cross‐sectional areas of the skeletal muscle, subcutaneous fat, and visceral fat were measured. Skeletal muscle mass status was defined based on the cut‐offs of skeletal muscle index (cm^2^/m^2^), calculated as the area of skeletal muscle divided by height squared. Overall survival was estimated using the Kaplan–Meier method, and differences in survival probabilities were compared using the log‐rank test. Cox proportional hazards regression analysis was conducted to determine the association with overall survival.

**Results:**

A total of 636 patients with a median age of 61 years (interquartile range, 54.0–68.5 years; 321 men and 315 women) were included. Obese patients (body mass index ≥ 25 kg/m^2^) had longer overall survival than non‐obese patients (mean, 110.2 months vs. 98.7 months; log‐rank *P* = 0.015). Under multivariable Cox proportional hazard regression analysis, obesity was associated with longer overall survival after adjusting for covariates (hazard ratio, 0.59; 95% confidence interval, 0.40–0.86; *P* = 0.007). The prognostic value of obesity remained and predicted favourable overall survival after additional adjusting for skeletal muscle mass status (hazard ratio, 0.57; 95% confidence interval, 0.36–0.89; *P* = 0.014), skeletal muscle index (hazard ratio, 0.53; 95% confidence interval, 0.33–0.84; *P* = 0.008), or skeletal muscle area (hazard ratio, 0.61; 95% confidence interval, 0.38–0.98; *P* = 0.041). No association was observed between skeletal muscle mass status and the impact of body mass index on overall survival (*P* for interaction = 0.512).

**Conclusions:**

Obesity was associated with favourable overall survival, independent of skeletal muscle mass, after surgical excision of lung adenocarcinoma.

## Introduction

Lung cancer is a leading cause of mortality, causing approximately 1.4 million deaths worldwide.^1^ Among the histological subtypes of non‐small‐cell lung cancer, which represents 85% of lung cancers, lung adenocarcinoma has become the most common subtype over the past three decades.^2^ In addition to the TNM stage, sex, smoking status, and other physical factors, including performance status, also influence postoperative prognosis.^3^

Obesity is widely recognized as a risk factor for cardiovascular disease,^4^ has been associated with an increased risk of developing and dying from most cancer types, and is considered to be a major preventable cause of cancer mortality.^5^ However, several observational studies have documented conflicting findings that obese patients exhibit better clinical outcomes in cardiovascular diseases, cancers, and other chronic diseases.^6^ This unexpected and paradoxical benefit of obesity, termed the ‘obesity paradox’, is a topic of immense interest and has also been reported in patients with lung cancer.^7^ Although methodological problems, such as reverse causality, collider‐stratification, or detection bias, other confounding factors, or inadequacy of body mass index (BMI) as an accurate representation of obesity, have been considered as possible explanations, the obesity paradox remains poorly understood.^8^

Sarcopenia, characterized by loss of skeletal muscle strength, quantity, or quality, is increasingly recognized as a factor in cancer cachexia syndrome.^9^ Several studies have reported its association with a poor prognosis, and it has emerged as a potential modifiable risk factor, as well as an important prognostic predictor in various types of cancers.^10^, ^11^ Using computed tomography (CT), which allows non‐invasive and objective simultaneous quantification of skeletal muscle, subcutaneous fat, and visceral fat, previous studies have reported that low skeletal muscle mass determined by cross‐sectional areas on CT is an independent prognostic factor which adversely impacts patients' survival in both small‐cell and non‐small‐cell lung cancers.^11^, ^12^ Considering that BMI does not differentiate between muscle and fat, it might be reasonable to assume that skeletal muscle mass could be a confounding factor in the obesity paradox. To our knowledge, however, there have been no studies examining the obesity paradox in the context of postoperative outcomes in patients with lung adenocarcinoma regarding its association with skeletal muscle mass.

In this study, we investigated the impact of obesity on postoperative outcomes in patients with lung adenocarcinoma after surgical excision. The prognostic value of obesity was explored to determine whether it is associated with skeletal muscle mass measured by cross‐sectional imaging.

## Methods

### Patients

**Figure 1:**
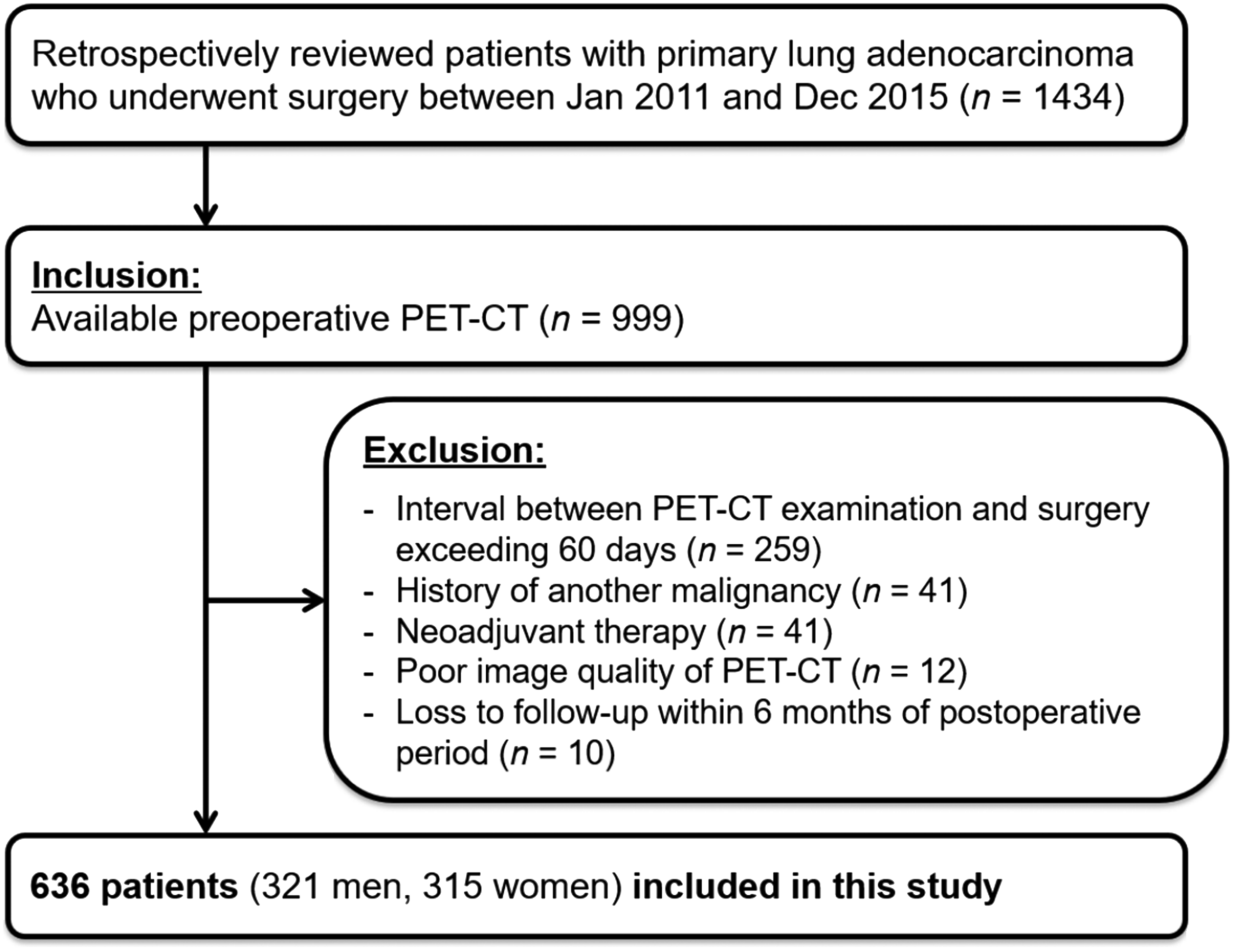
Flow diagram illustrating selection of the study population. PET‐CT, positron emission tomography‐computed tomography.

Our institutional review board (Samsung Medical Center, IRB file No. 2021‐03‐141) approved this retrospective study and waived the requirement for informed consent. The study was performed in accordance with the Declaration of Helsinki. We reviewed the electronic medical records of 1434 patients who underwent thoracic surgery between January 2011 and December 2015 for initially and pathologically diagnosed primary lung adenocarcinoma. Among them, 999 patients who had available preoperative positron emission tomography‐computed tomography (PET‐CT) were enrolled. Thereafter, the following exclusion criteria were applied, (i) interval between PET‐CT examination and surgery exceeding 60 days (*n* = 259), (ii) history of another malignancy (*n* = 41), (iii) patients who underwent neoadjuvant therapy (*n* = 41), (iv) poor image quality of PET‐CT (e.g. metallic or streaking artefact; *n* = 12), and (v) loss to follow‐up within 6 months of the postoperative period (*n* = 10) (*Figure* 1).

### PET‐CT examination

Whole‐body PET and non‐contrast CT scans were acquired using an integrated PET‐CT scanner (Discovery STE, GE Healthcare, Milwaukee, WI, USA). Whole‐body CT images were obtained using a 16‐slice helical CT scanner with a tube voltage of 140 kVp, tube current of 30–170 mAs, pitch of 1.75, and section width of 3.75 mm. Thereafter, emission scans were performed from the thigh to the head for 2.55 min per frame about 60 min after the intravenous administration of [^18^F] fluorodeoxyglucose (5.5 MBq/kg body weight).

### Image analysis

**Figure 2:**
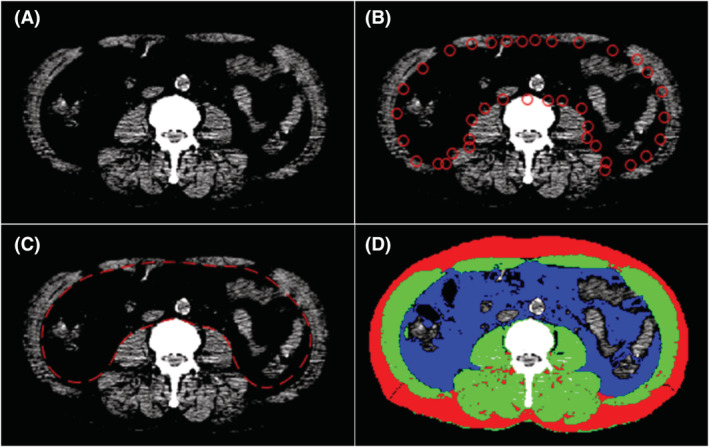
An example of semiautomatic quantification of body composition in a 56‐year‐old man with lung adenocarcinoma. CT image at the third lumbar vertebra level after linear transformation of the intensity into 0 to +100 Hounsfield unit (A). After semiautomatic manipulation (B), the boundary between the muscles and the inner tissues was detected (C). Pixels of fat and muscle were identified, measuring the cross‐sectional areas of the muscle, subcutaneous fat, and visceral fat to be 168.6, 124.9, and 146.6 cm^2^, respectively (D). CT, computed tomography.

A board‐certified radiologist with 6 years of experience of musculoskeletal imaging determined the areas of skeletal muscle, subcutaneous fat, and visceral fat, blinded to patient information. The non‐contrast CT images of the PET‐CT studies were analysed using an in‐house open‐source software (BMI_CT, available at https://sourceforge.net/projects/muscle‐fat‐area‐measurement/) based on MATLAB version R2010a (Mathworks Inc., Natick, MA, USA). At the level of the third lumbar vertebrae,^13^ cross‐sectional areas (cm^2^) of skeletal muscle including the rectus, transverse and oblique abdominal muscles, psoas muscles, paraspinal muscles, subcutaneous fat, and visceral fat were measured using a semiautomated method. (i) To highlight the muscle boundary, the intensity of the CT image was linearly transformed into 0 to +100 Hounsfield units (HU). (ii) After semiautomatic manipulation, the boundary between the muscles and the inner tissues was detected using the active contour method by minimizing a cost function, dividing CT images into inner and outer regions. (iii) Pixels in the fat and muscle were then identified using cut‐off values of −300 to −50 HU and −29 to +150 HU, respectively (*Figure* 2).

The skeletal muscle area (cm^2^) was normalized by dividing by the square of the height (m^2^) of the patient to calculate skeletal muscle index (SMI)^14^; low skeletal muscle mass was defined as a SMI of ≤52.4 cm^2^/m^2^ in men and ≤38.5 cm^2^/m^2^ in women, as proposed by a CT‐based study of patients with cancer.^15^ Total fat area was defined as the sum of the areas of subcutaneous fat and visceral fat. The visceral‐to‐subcutaneous fat ratio (VSR) was calculated by dividing the area of visceral fat by that of subcutaneous fat.

### Clinical variables and endpoints

For clinical data, electronic medical records were reviewed; data regarding age, sex, smoking status, the Charlson comorbidity index,^16^ the Eastern Cooperative Oncology Group performance status, height, and body weight before the day of surgery were collected. We also recorded the pathologic stage according to the 7th edition of the TNM classification for lung cancer,^17^ type of surgery, and whether the patients underwent adjuvant therapy. BMI was calculated as the weight divided by height squared (kg/m^2^) and categorized according to criteria for Asia‐Pacific classification of underweight (<18.5 kg/m^2^), normal (18.5–22.9 kg/m^2^), overweight (23.0–24.9 kg/m^2^), or obese (≥25 kg/m^2^).^18^ We also recorded the date of surgery, date of death, or date of last follow‐up to calculate the overall survival (OS) as the primary endpoint of this study, which was defined as the time from surgery to death from any cause. The secondary endpoint was relapse‐free survival (RFS), defined as the time from surgery to locoregional recurrence, distant metastasis, or death from any cause, whichever occurs first. Patients without an event were censored at the last follow‐up for both OS and RFS.

### Statistical analysis

Patient characteristics were compared between the obese and non‐obese groups. Continuous variables were compared using the Wilcoxon rank‐sum test, and categorical variables were compared using the chi‐squared or Fisher's exact test.

The Kaplan–Meier method with the log‐rank test was used to characterize event‐time distributions, and OS and RFS according to BMI and skeletal muscle mass status were evaluated. We used the Cox proportional hazards model to estimate the hazard ratios and corresponding 95% confidence intervals (CIs) for the risk of mortality and recurrent/metastatic disease associated with skeletal muscle mass status, SMI, skeletal muscle area, BMI, subcutaneous fat area, visceral fat area, total fat area, and VSR; BMI was treated both categorically (underweight/normal/overweight/obese) and continuously (per standard deviation), whereas other variables, except for skeletal muscle mass status, were treated continuously (per standard deviation). Adjustments for covariates were performed with and without adjustment for muscle‐derived variables. We first adjusted for age at surgery (continuously per year), sex (men/women), type of surgery (wedge resection/segmental resection/lobectomy/pneumonectomy), pathologic stage (I/II/III/IV), the Eastern Cooperative Oncology Group performance status (0/≥1), smoking status (never smoker/current or ex‐smoker), the Charlson comorbidity index (continuously per point), and adjuvant therapy (no/yes). Thereafter, BMI, subcutaneous fat area, visceral fat area, total fat area, and VSR were additionally adjusted for skeletal muscle mass status (normal/low), SMI (continuously per cm^2^/m^2^), and skeletal muscle area (continuously per cm^2^), to explore whether their prognostic value depended on muscle quantity. The interaction term in the Cox proportional hazard regression models adjusting for covariates was used to determine whether the association between BMI and OS differed between men and women, or between patients with low and normal skeletal muscle mass, with BMI as a categorical variable.

All statistical analyses were performed using SPSS Statistics (Version 27.0; IBM Corp., Armonk, NY, USA) and MedCalc® Statistical Software version 20.009 (MedCalc Software Ltd, Ostend, Belgium). Statistical significance was set at *P* < 0.05.

## Results

A total of 636 patients (median age, 61.0 years; interquartile range [IQR], 54.0–68.5 years; 321 men and 315 women) were included in the analysis. The median interval between the PET‐CT examination and surgery was 14.0 days (IQR, 9.0–23.0 days). According to the cut‐offs, 264 (41.5%) patients had low skeletal muscle mass. Our cohort consisted of 13 underweight patients (2.0%), 205 patients (32.2%) with normal BMI, 201 overweight patients (31.6%), and 217 patients (34.1%) with obesity, 21 (3.3%) of whom had a BMI exceeding 30 kg/m^2^. During the postoperative follow‐up period, with a median of 70.0 months (IQR, 42.5–85.0 months), 177 patients (27.8%) had died, three of whom died from postoperative complications during hospitalization. Locoregional recurrence and/or distant metastases occurred in 257 patients (40.4%).

Baseline patient characteristics are shown in *Table* 1, providing a comparison between obese and non‐obese patients, including patients who were underweight, had normal BMI, or were overweight. Obese patients had a higher BMI (median, 26.4 kg/m^2^ vs. 22.9 kg/m^2^, *P* < 0.001), lower prevalence of low skeletal muscle mass (13.3% vs. 56.1%, *P* < 0.001), higher SMI (median, 50.8 cm^2^/m^2^ vs. 44.2 cm^2^/m^2^, *P* < 0.001), skeletal muscle area (median, 128.9 cm^2^ vs. 113.7 cm^2^, *P* < 0.001), subcutaneous fat area (median, 156.9 cm^2^ vs. 109.5 cm^2^, *P* < 0.001), visceral fat area (median, 139.5 cm^2^ vs. 78.8 cm^2^, *P* < 0.001), total fat area (median, 304.8 cm^2^ vs. 200.9 cm^2^, *P* < 0.001), and VSR (median, 0.87 vs. 0.78, *P* = 0.002) than non‐obese patients. There were no other significant differences between the characteristics of obese and non‐obese patients.

**Table 1:** Baseline characteristics

Characteristic	Total (*n* = 636)	Non‐obese^e^ (*n* = 419)	Obese (*n* = 217)	*P*
Age at surgery (years)^a^	61.0 [54.0, 68.5]	60.0 [53.0, 68.8]	62.0 [56.0, 68.3]	0.106
Male sex, *n* (%)^b^	321 (50.5%)	217 (51.8%)	104 (47.9%)	0.356
BMI (kg/m^2^)^a^	24.1 [22.2, 25.6]	22.9 [21.4, 24.0]	26.4 [25.6, 27.7]	<0.001
Low skeletal muscle mass, *n* (%)^b^ ^,^ ^c^	264 (41.5%)	235 (56.1%)	29 (13.3%)	<0.001
SMI (cm^2^/m^2^)^a^	46.1 [41.5, 51.0]	44.2 [39.7, 48.9]	50.8 [44.6, 55.4]	<0.001
Skeletal muscle area (cm^2^) ^a^	117.6 [101.3, 140.1]	113.7 [98.7, 133.7]	128.9 [107.9, 155.5]	<0.001
Subcutaneous fat area (cm^2^) ^a^	124.7 [95.5, 158.2]	109.5 [86.1, 137.6]	156.9 [128.5, 198.7]	<0.001
Visceral fat area (cm^2^)^a^	97.9 [63.4, 139.3]	78.8 [52.1, 116.8]	139.5 [101.2, 170.7]	<0.001
Total fat area (cm^2^)^a^	234.9 [177.6, 289.8]	200.9 [150.6, 247.6]	304.8 [263.3, 352.1]	<0.001
VSR^a^	0.81 [0.53, 1.08]	0.78 [0.50, 1.04]	0.87 [0.62, 1.16]	0.002
Current/ex‐smoker, *n* (%)^c^	290 (45.6%)	195 (46.5%)	95 (43.8%)	0.508
Type of surgery, *n* (%)^d^				0.800
Wedge resection^c^	25 (3.9%)	16 (3.8%)	9 (4.1%)	0.840
Segmental resection^c^	24 (3.8%)	16 (3.8%)	8 (3.7%)	0.934
Lobectomy^c^	580 (91.2%)	381 (90.9%)	199 (91.7%)	0.744
Pneumonectomy^d^	7 (1.1%)	6 (1.4%)	1 (0.4%)	0.432
ECOG PS ≥ 1, *n* (%)^c^	157 (24.7%)	109 (27.3%)	48 (23.0%)	0.294
Adjuvant therapy, *n* (%)^c^	234 (36.8%)	148 (37.0%)	86 (41.1%)	0.318
Charlson comorbidity index ≥1, *n* (%)^c^	157 (24.7%)	101 (24.1%)	56 (25.8%)	0.376
Pathologic stage, *n* (%)^c^				0.949
I^c^	368 (57.9%)	241 (57.5%)	127 (58.5%)	0.807
II^c^	129 (20.3%)	84 (20.0%)	45 (20.7%)	0.838
III^c^	112 (17.6%)	75 (17.9%)	37 (17.1%)	0.790
IV^c^	27 (4.2%)	19 (4.5%)	8 (3.7%)	0.615

BMI, body mass index; ECOG PS, Eastern Cooperative Oncology Group performance status; SMI, skeletal muscle index; VSR, visceral‐to‐subcutaneous fat ratio.

^a^ Wilcoxon rank‐sum test. Numbers are shown as medians and interquartile ranges in square brackets.

^b^ Defined as a SMI of ≤52.4 cm^2^/m^2^ in men and ≤38.5 cm^2^/m^2^ in women.

^c^ Chi‐squared test.

^d^ Fischer's exact test.

^e^ Including patients who were underweight, having normal BMI, and who were overweight.

### Overall survival

In all patients, mean OS was 103.5 months (95% CI, 99.6–107.3 months), and the median OS was not reached. The number of events in patients who were underweight, having normal BMI, overweight, and obese were 8 (61.5%), 61 (29.8%), 61 (30.4%), and 47 (21.7%), respectively, whereas those in patients with normal‐ and low skeletal muscle mass were 85 (22.9%) and 92 (34.9%), respectively.

**Figure 3:**
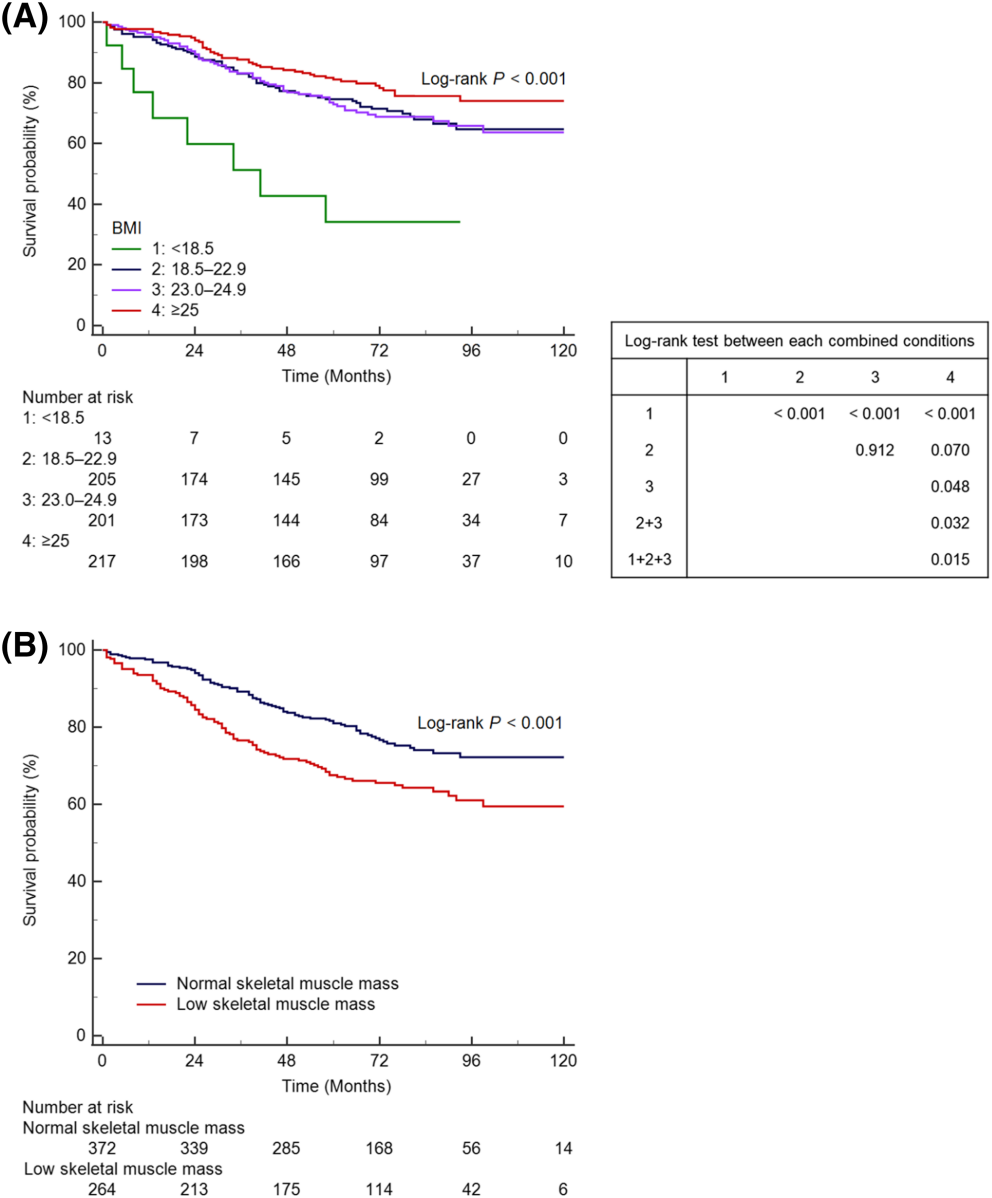
Kaplan–Meier estimates of overall survival, according to BMI categories (A) and skeletal muscle mass status (B). BMI, body mass index.

Kaplan–Meier curves and log‐rank tests showed that OS differed significantly between the BMI categories (log‐rank *P* < 0.001), with poor OS in underweight patients (mean OS, 47.2 months; 95% CI, 26.9–67.6 months) compared with patients who had normal BMI (mean OS, 98.8 months; 95% CI, 92.2–105.4 months), those who were overweight (mean OS, 99.8 months; 95% CI, 93.0–106.6 months), or those who were obese (mean OS, 110.2 months; 95% CI, 104.2–116.3 months) (log‐rank *P* values, <0.001); OS of obese patients was significantly longer than that of non‐obese patients, both before (mean OS, 98.7 months; 95% CI, 93.9–103.5 months; log‐rank *P* = 0.015) and after (mean OS, 99.9 months, 95% CI, 95.1–104.7 months; log‐rank *P* = 0.032), excluding underweight patients, and tended to be longer than that of patients with normal BMI (mean OS, 98.8 months; 95% CI, 92.2–105.4 months; log‐rank *P* = 0.070) (*Figure* 3A). Patients with low skeletal muscle mass (mean OS, 93.3 months; 95% CI, 87.1–99.6 months) had shorter OS than patients with normal skeletal muscle mass (mean OS, 109.4 months; 95% CI, 104.8–114.0 months) (log‐rank *P* < 0.001) (*Figure* 3B).

In multivariable Cox proportional hazard regression models, before adjusting for muscle‐derived variables, a high skeletal muscle area was associated with favourable prognosis, demonstrating a 24% decreased risk of death as skeletal muscle area increased by 1 standard deviation (24.9 cm^2^). Compared with patients with normal BMI, obese patients had a 41% decreased risk of death, while underweight patients had a 171% increased risk of death. The risk of death decreased by 21% as BMI increased by 1 standard deviation (2.9 kg/m^2^). An increase of 1 standard deviation in visceral fat area (53.9 cm^2^) and total fat area (87.3 cm^2^) was also associated with 20% deceased risk of death. In contrast, low skeletal muscle mass, SMI, subcutaneous fat area, and VSR were not significantly associated with OS (*Table* 2; Model I). The association observed in BMI (continuous and categorical), visceral, and total fat area remained significant after additional adjustment for skeletal muscle mass status and SMI (*Table* 2; Models IIa and IIb). Among them, only the underweight and obese BMI categories remained significant when adjusted for skeletal muscle area, with a 165% increased and 39% decreased risk of death in underweight and obese patients, respectively (*Table* 2; Model IIc).

**Table 2:** Association between muscle‐derived variables, BMI, adiposity, and overall survival

Characteristic	Model I		Model IIa^a^		Model IIb^b^		Model IIc^c^	
	HR (95% CI)	*P*	HR (95% CI)	*P*	HR (95% CI)	*P*	HR (95% CI)	*P*
Skeletal muscle mass status								
Normal	1 [reference]							
Low^d^	1.31 (0.95–1.82)	0.103						
SMI (cm^2^/m^2^)								
Continuously, per SD (7.0)	0.87 (0.73–1.03)	0.101						
Skeletal muscle area (cm^2^)								
Continuously, per SD (24.9)	0.76 (0.60–0.95)	0.016						
BMI (kg/m^2^)								
Underweight (<18.5)	2.71 (1.24–5.93)	0.012	2.74 (1.25–6.02)	0.012	2.96 (1.31–6.68)	0.009	2.65 (1.19–5.88)	0.017
Normal (18.5–22.9)	1 [reference]		1 [reference]		1 [reference]		1 [reference]	
Overweight (23.0–24.9)	0.83 (0.57–1.20)	0.316	0.82 (0.56–1.20)	0.304	0.79 (0.53–1.16)	0.226	0.85 (0.57–1.25)	0.400
Obese (≥25)	0.59 (0.40–0.86)	0.007	0.57 (0.36–0.89)	0.014	0.53 (0.33–0.84)	0.008	0.61 (0.38–0.98)	0.041
Continuously, per SD (2.9)	0.79 (0.67–0.93)	0.005	0.79 (0.65–0.96)	0.020	0.76 (0.61–0.96)	0.019	0.83 (0.66–1.03)	0.097
Subcutaneous fat area (cm^2^)								
Continuously, per SD (52.4)	0.83 (0.68–1.00)	0.052	0.85 (0.70–1.04)	0.114	0.86 (0.70–1.06)	0.152	0.90 (0.73–1.11)	0.329
Visceral fat area (cm^2^)								
Continuously, per SD (53.9)	0.80 (0.70–0.93)	0.005	0.81 (0.69–0.96)	0.014	0.81 (0.68–0.96)	0.018	0.84 (0.70–1.00)	0.053
Total fat area (cm^2^)								
Continuously, per SD (87.3)	0.80 (0.68–0.93)	0.005	0.81 (0.69–0.96)	0.016	0.81 (0.68–0.97)	0.021	0.84 (0.70–1.01)	0.070
VSR								
Continuously, per SD (0.60)	0.87 (0.72–1.06)	0.163	0.88 (0.73–1.07)	0.208	0.89 (0.73–1.08)	0.226	0.90 (0.74–1.09)	0.291

BMI, body mass index; CI, confidence interval; HR, hazard ratio; SD, standard deviation; SMI, skeletal muscle index; VSR, visceral‐to‐subcutaneous fat ratio.

All models were adjusted for the following covariates: age at surgery (continuous per year), sex (men/women), type of surgery (wedge resection/segmental resection/lobectomy/pneumonectomy), pathologic stage (I/II/III/IV), Eastern Cooperative Oncology Group performance status (0/≥1), smoking status (never smoker/current or ex‐smoker), Charlson comorbidity index (continuous per point), and adjuvant therapy (no/yes).

^a^ Model IIa was adjusted for covariates plus skeletal muscle mass status (normal/low; defined as a SMI of ≤52.4 cm^2^/m^2^ in men and ≤38.5 cm^2^/m^2^ in women).

^b^ Model IIb was adjusted for covariates plus skeletal muscle index in cm^2^/m^2^ (continuous).

^c^ Model IIc was adjusted for covariates plus skeletal muscle area in cm^2^ (continuous).

^d^ Defined as a SMI of ≤52.4 cm^2^/m^2^ in men and ≤38.5 cm^2^/m^2^ in women.

### Interactions of body mass index with sex and skeletal muscle mass status

The association between BMI and OS did not reach statistical significance in differences between men and women when BMI was categorized into four subgroups of underweight, normal, overweight, or obese (*P* for interaction = 0.295), or into two subgroups of obese and others (*P* for interaction > 0.050). The association of BMI with OS was similar between patients between normal and low skeletal muscle mass (*P* for interaction = 0.512). Likewise, the association of obesity with OS did not significantly differ between patients between normal and low skeletal muscle mass (*P* for interaction > 0.05) (*Table* 3).

**Table 3:** Interaction of BMI with sex and skeletal muscle mass status for overall survival

Characteristic	Sex^a^		*P* for interaction	Skeletal muscle mass status^b^		*P* for interaction
	Men	Women		Normal	Low^c^	
	HR (95% CI)	HR (95% CI)		HR (95% CI)	HR (95% CI)	
BMI (kg/m^2^)			0.295			0.512
Underweight (<18.5)	1.62 (0.57–4.58)	4.81 (1.25–18.43)		NA	2.86 (1.17–6.97)	
Normal (18.5–22.9)	1 [reference]	1 [reference]		1 [reference]	1 [reference]	
Overweight (23.0–24.9)	0.78 (0.48–1.26)	0.64 (0.30–1.34)		0.61 (0.33–1.12)	0.78 (0.48–1.27)	
Obese (≥25)	0.54 (0.28–1.02)	0.50 (0.23–1.11)		0.47 (0.26–0.84)	0.61 (0.28–1.33)	
Obese						
vs. Underweight + normal + overweight	0.67 (0.39–1.13)	0.64 (0.35–1.19)	0.209	0.64 (0.41–1.01)	0.68 (0.32–1.42)	0.861
vs. Normal + overweight	0.64 (0.38–1.08)	0.66 (0.35–1.23)	0.161	0.64 (0.41–1.01)	0.73 (0.35–1.54)	0.715
vs. Normal	0.54 (0.25–1.17)	0.44 (0.19–1.02)	0.103	0.45 (0.25–0.80)	0.68 (0.30–1.53)	0.320

BMI, body mass index; CI, confidence interval; HR, hazard ratio; NA, not available.

^a^ Adjusted for age at surgery (continuous per year), type of surgery (wedge resection/segmental resection/lobectomy/pneumonectomy), pathologic stage (I/II/III/IV), Eastern Cooperative Oncology Group performance status (0/≥1), smoking status (never smoker/current or ex‐smoker), Charlson comorbidity index (continuous per point), adjuvant therapy (no/yes), and skeletal muscle area (continuous per cm^2^).

^b^ Adjusted for age at surgery (continuous per year), sex (men/women), type of surgery (wedge resection/segmental resection/lobectomy/pneumonectomy), pathologic stage (I/II/III/IV), Eastern Cooperative Oncology Group performance status (0/≥1), smoking status (never smoker/current or ex‐smoker), Charlson comorbidity index (continuous per point), and adjuvant therapy (no/yes).

^c^ Defined as a SMI of ≤52.4 cm^2^/m^2^ in men and ≤38.5 cm^2^/m^2^ in women.

### Relapse‐free survival

In all patients, median RFS was 77.0 months (95% CI, 65.0–132.0 months) and the number of events was 304 (47.9%), including locoregional recurrence/distant metastasis (*n* = 257) and death without locoregional recurrence/distant metastasis (*n* = 47); the number of events in patients who were underweight, having normal BMI, overweight, and obese were 5 (38.5%), 96 (46.8%), 101 (50.3%), and 102 (47.0%), respectively, whereas those in patients with normal and low skeletal muscle mass were 176 (47.3%) and 128 (48.5%), respectively.

**Figure 4:**
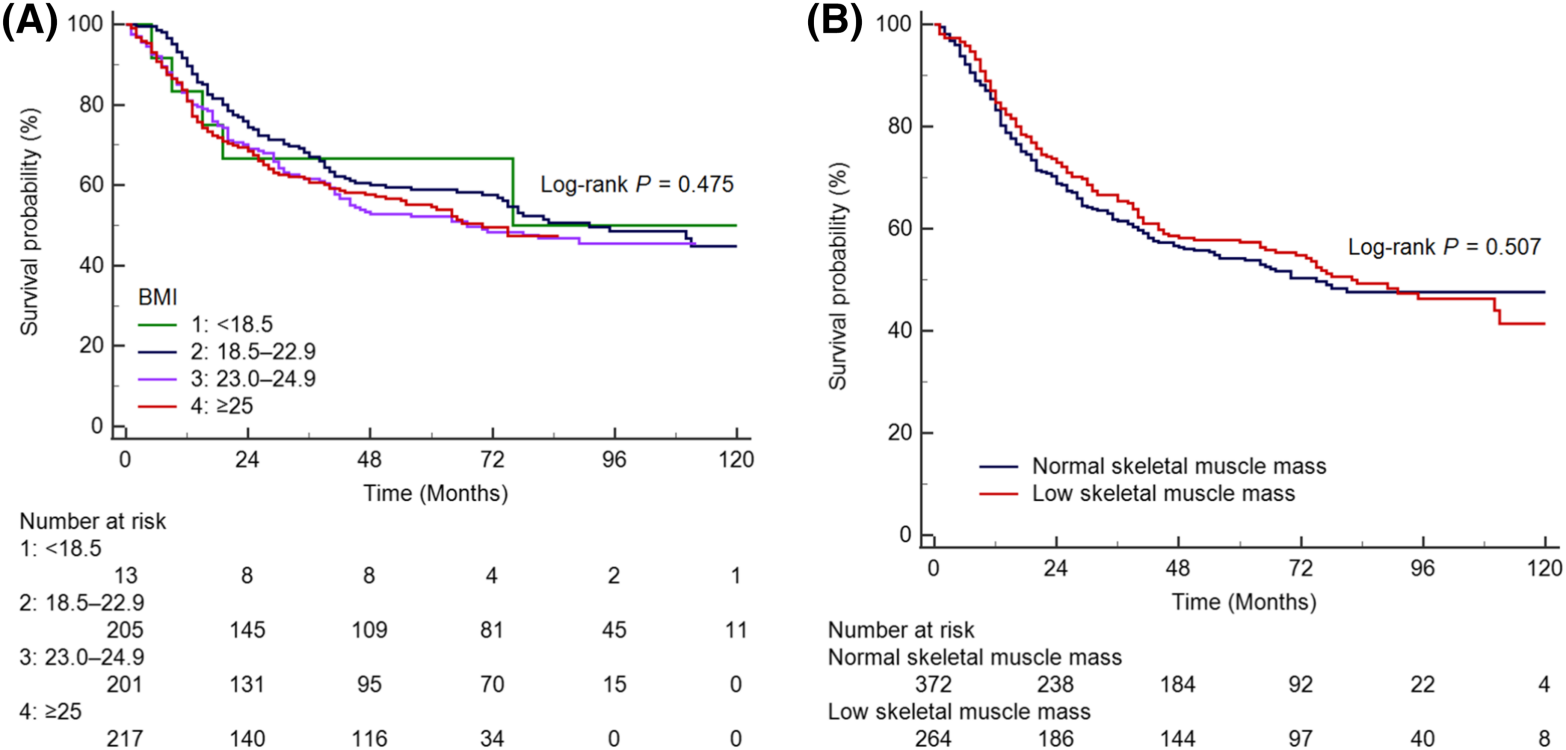
Kaplan–Meier estimates of relapse‐free survival, according to BMI categories (A) and skeletal muscle mass status (B). BMI, body mass index.

Kaplan–Meier curves and log‐rank tests showed that RFS was not significantly different between the BMI categories (log‐rank *P* = 0.475) (*Figure* 4A) or skeletal muscle mass status (log‐rank *P* = 0.507) (*Figure* 4B). None of the muscle‐derived variables, BMI, and adiposity was associated with RFS in multivariable Cox proportional hazard regression model (*Table* 4).

**Table 4:** Association between muscle‐derived variables, BMI, adiposity, and relapse‐free survival

Characteristic	HR (95% CI)	*P*
Skeletal muscle mass status		
Normal	1 [reference]	
Low^a^	0.95 (0.74–1.23)	0.704
SMI (cm^2^/m^2^)		
Continuously, per SD (7.0)	0.99 (0.86–1.13)	0.857
Skeletal muscle area (cm^2^)		
Continuously, per SD (24.9)	0.93 (0.77–1.11)	0.402
BMI (kg/m^2^)		
Underweight (<18.5)	1.02 (0.41–2.56)	0.969
Normal (18.5–22.9)	1 [reference]	
Overweight (23.0–24.9)	1.27 (0.95–1.69)	0.103
Obese (≥25)	1.26 (0.94–1.67)	0.120
Continuously, per SD (2.9)	1.03 (0.92–1.15)	0.614
Subcutaneous fat area (cm^2^)		
Continuously, per SD (52.4)	0.99 (0.87–1.12)	0.860
Visceral fat area (cm^2^)		
Continuously, per SD (53.9)	0.99 (0.88–1.12)	0.917
Total fat area (cm^2^)		
Continuously, per SD (87.3)	0.99 (0.88–1.11)	0.875
VSR		
Continuously, per SD (0.60)	0.99 (0.84–1.17)	0.919

BMI, body mass index; CI, confidence interval; HR, hazard ratio; SD, standard deviation; SMI, skeletal muscle index; VSR, visceral‐to‐subcutaneous fat ratio.

Adjusted for the following covariates: age at surgery (continuous per year), sex (men/women), type of surgery (wedge resection/segmental resection/lobectomy/pneumonectomy), pathologic stage (I/II/III/IV), Eastern Cooperative Oncology Group performance status (0/≥1), smoking status (never smoker/current or ex‐smoker), Charlson comorbidity index (continuous per point), and adjuvant therapy (no/yes).

^a^ Defined as a SMI of ≤52.4 cm^2^/m^2^ in men and ≤38.5 cm^2^/m^2^ in women.

## Discussion

Our analyses, with long‐term follow‐up of patients with lung adenocarcinoma, demonstrated that BMI was a significant prognostic marker affecting survival after surgical excision, with patients having a BMI indicative of being underweight or obese being associated with poor and improved OS, respectively. These associations were independent of covariates, including age, sex, type of surgery, the Charlson comorbidity index, and pathologic stage. Although the survival advantage of obesity has already been reported in lung cancer,^7^ it is unclear whether this phenomenon exists irrespective of skeletal muscle mass. In contrast, the strength of our study was that it showed that the prognostic significance of obesity was independent of muscle‐derived variables such as skeletal muscle mass status, which were previously suspected to drive the obesity paradox. Likewise, the relationship between BMI and OS did not vary according to skeletal muscle mass status. In contrast, RFS did not differ significantly according to BMI or other body composition features.

As BMI does not distinguish between lean body mass and fat, the imperfection of BMI as a surrogate measure for obesity was considered convincing among several suggested hypotheses to address the obesity paradox.^19^ The study by Gonzalez *et al*.,^20^ demonstrating that cancer patients with excess fat were misclassified as having normal BMI, was one of the studies used to draw this theory. Moreover, they also argued that the association between obesity and higher survival rates was present only when the fat‐free mass index determined by bioelectrical impedance analysis was normal, encouraging the assessment of body composition in cancer patients. Since then, there have been accumulating studies explaining skeletal muscle mass status as a missing link between obesity and improved clinical outcomes.^21^, ^22^ However, our findings in patients with lung adenocarcinoma contrast with those of previous studies, showing that obesity is associated with improved OS, despite taking the quantity of skeletal muscle into consideration, and that there was no significant interaction between obesity and skeletal muscle mass status with respect to the impact on OS. Recently, Xu *et al*.^23^ also reported that obesity was associated with reduced mortality in patients with metastatic or castrate‐resistant prostate cancer, independent of CT‐determined sarcopenia and myosteatosis, which was comparable with the results of our study. Although residual confounding or reverse causation due to cancer cachexia^24^ could also be possible explanations for the obesity paradox, we tried to minimize their effects by adjusting for relevant covariates. Furthermore, in this study, obese and non‐obese patients did not differ in terms of variables other than BMI and body composition indices. Meanwhile, whether the obesity paradox exists independent of skeletal muscle quantity also in patients with other type of lung cancers, or patients who receive palliative chemotherapy requires further investigation as this study focused exclusively on the postoperative outcome in patients with lung adenocarcinoma; this association may especially differ in small‐cell lung cancer or squamous cell carcinoma where stronger smoker predominancy is noted, given that smoking may be an important factor leading to confounding and/or collider‐stratification bias.^8^ Regarding the adverse impact of an underweight BMI on OS, our study results were in line with a previous study on patients with lung cancer.^25^

The vast majority of previous retrospective studies on CT‐determined sarcopenia adopted SMI as a surrogate for sarcopenia,^26^ and so did this study. In 2018, however, the European Working Group on Sarcopenia in Older People clarified that the presence of low muscle strength is essential in diagnosing sarcopenia.^9^ Although this working group acknowledged that the third lumbar vertebra imaging by CT is an alternative validated tool for assessing muscle quantity, none of measurements based on CT, including SMI, are sufficient to make a diagnosis of sarcopenia, given that muscle quantity does not fully represent muscle function. In this regard, whether the obesity paradox still exists irrespective of sarcopenia confirmed by both low muscle strength as well as quantity or quality would be worth investigation, which may require future prospective study.

Interestingly, we observed that an increase in fat quantity as well as BMI was related to improved OS, contrary to their previously described association with cardiometabolic risk.^27^ Although the beneficial influence of adiposity has been presumed to be attributed to their protectiveness with respect to muscle loss,^28^ our study showed contrasting results, indicating that the association between adiposity and OS was independent of skeletal muscle mass status and SMI. Notably, there was a tendency for visceral adiposity, rather than subcutaneous adiposity, to be associated with improved OS, which was also comparable with results of previous studies by Xu *et al*.^23^ and Hong *et al*.^29^ While fat accumulation can occur in both subcutaneous and visceral regions, the term ‘obesity’ mainly reflects visceral fat accumulation.^30^ Subcutaneous and visceral fat are also functionally different from each other, and the latter is suggested to be associated with increased proinflammatory cytokines, chronic inflammation, and carcinogenesis.^31^, ^32^ Despite the adverse impact of obesity on immune dysfunction, a previous study hypothesized that heightened anti‐tumour efficacy of checkpoint blockade in obese patients could be a contributing factor to the obesity paradox.^33^ However, this theory is not likely to be applied to our study on the postoperative outcomes of lung cancer patients. Considering that obesity and visceral adiposity was associated with improved OS despite absence of RFS gain, improved energy reserves or tumour‐suppressing effects produced by adipose tissues in patients with ‘metabolically healthy obesity’^34^ after recurrent disease could be one of the possible explanations, although presumptive. Future studies are warranted to validate these hypotheses and elucidate the true significance of visceral adiposity.

As all the patients were Asians, we adopted the BMI criteria for the Asia‐Pacific classification^18^ among several suggested cut‐off points for BMI classification. To the best of our knowledge, no study has explored the obesity paradox in lung cancer based on this BMI criteria. Although the definitions of obesity differed, our result is in line with that of a meta‐analysis reporting an independent protective association between premorbid obesity and lung cancer‐related mortality,^35^ as the majority of obese patients in our study, with BMI of ≥25 kg/m^2^, would be classified as overweight according to the standard WHO definition.^36^ Considering that mortality curves for BMI are usually U‐shaped, with increasing mortality at both ends,^37^ the question of whether the survival benefit from obesity and visceral adiposity is independent of muscle persists in even patients with higher BMI, including morbid obesity, and may require further investigation. This was considered to be one of the limitations of this study. Moreover, whether the obesity paradox exists independent of skeletal muscle quantity in non‐Asians is another topic for future research.

This study had several limitations. First, there could be a selection bias inherent to this retrospective study conducted in a single tertiary hospital. In particular, the relatively favourable prognosis in the study population was probably attributed to the large proportion of early‐stage patients, as well as the fact that the majority of obese patients were premorbid, as described above, might have influenced our study results. Second, the causal relationship between obesity and OS could not be clearly determined in this observational study. Although adjusting for clinically relevant covariates could mitigate the influence of reverse causality, it might not have been eliminated. In addition, the presence of unmeasured or residual confounding factors may have been possible. Third, longitudinal changes in body composition or BMI were not evaluated, although they have been reported to be associated with patient survival.^38^ Many patients did not have postoperative body weight records or PET‐CT follow‐up examinations, making such analysis difficult. A subsequent prospective study with longitudinal measurements would be beneficial to confirm our study results. Fourth, this study focused only on the muscle quantity to explain the obesity paradox, excluding serologic tests such as C‐reactive protein or albumin. Given that visceral fat composition is recently suggested to be associated with inflammatory response and explains the obesity paradox,^39^ further research is demanded. Lastly, the lack of a validation sample is another limitation in this study.

In conclusion, obesity was associated with improved long‐term OS after surgical excision of lung adenocarcinoma, independent of skeletal muscle mass. The cross‐sectional area of visceral fat also tended to be associated with OS when the body composition was analysed. Our study results imply that BMI should be considered as a prognostic marker that can be easily assessed, despite its limitations. Future studies should investigate the true prognostic significance of BMI and visceral fat and define the mechanism that links BMI and patient survival.

## Conflict of interest

All the authors declare no potential conflicts of interests.
